# Birth characteristics and childhood leukemia in Switzerland: a register-based case–control study

**DOI:** 10.1007/s10552-021-01423-3

**Published:** 2021-04-20

**Authors:** Judith E. Lupatsch, Christian Kreis, Garyfallos Konstantinoudis, Marc Ansari, Claudia E. Kuehni, Ben D. Spycher

**Affiliations:** 1 Institute of Social and Preventive Medicine (ISPM), University of Bern, Mittelstrasse 43, 3012 Bern, Switzerland; 2 Institute of Pharmaceutical Medicine, University of Basel, Basel, Switzerland; 3 MRC Centre of Environment and Health, Department of Epidemiology and Biostatistics, School of Public Health, Imperial College London, London, UK; 4 CANSEARCH Research Laboratory, Department of Paediatrics, Gynaecology and Obstetrics, Geneva University, Geneva, Switzerland; 5 Onco-Hematology Unit, Department of Women, Child and Adolescent, Geneva University Hospital, Geneva, Switzerland

**Keywords:** Epidemiology, Birth weight, Maternal age, Paternal age, Parity, Twins

## Abstract

**Purpose:**

Initial genetic alterations in the development of childhood leukemia occur in utero or before conception; both genetic and environmental factors are suspected to play a role. We aimed to investigate the associations between childhood leukemia and perinatal characteristics including birth order, birth interval to older siblings, parental age, birth weight, and multiple birth.

**Methods:**

We identified cases diagnosed between 1981 and 2015 and born in Switzerland between 1969 and 2015 from the Swiss Childhood Cancer Registry and randomly sampled five controls per case from national birth records matched on date of birth, sex, and municipality of residence at birth. We used conditional logistic regression to investigate associations between perinatal characteristics and leukemia at ages 0–15 and 0–4 years, and the subtypes acute lymphoblastic leukemia (ALL) and acute myeloid leukemia (AML).

**Results:**

The study included 1,403 cases of leukemia. We observed increased risks associated with high birth weight (adjusted OR 1.37, 95% CI 1.12–1.69) and multiple birth (1.89, 1.24–2.86). These associations were similar for ALL and stronger for leukemia at ages 0–4 years. For AML, we observed an increased risk for higher birth order (3.08, 0.43–22.03 for fourth or later born children). We found no associations with other perinatal characteristics.

**Conclusion:**

This register-based case–control study adds to the existing evidence of a positive association between high birth weight and risk of childhood leukemia. Furthermore, it suggests children from multiple births are at an increased risk of leukemia.

**Supplementary Information:**

The online version contains supplementary material available at 10.1007/s10552-021-01423-3.

## Introduction

Both genetic and environmental factors are suspected to play a role in the etiology of childhood leukemia [1, 2]. Established risk factors include ionizing radiation in medium to high doses and certain inherited disorders such as Li Fraumeni syndrome or chromosomal abnormalities such as Down syndrome, yet these account for only a fraction of cases [2, 3]. Suspected environmental risk factors include low level ionizing radiation [4, 5], air pollution [6], benzene [7], exposure to infections [8, 9], agricultural pesticides [10, 11], or parental occupational exposures [12, 13]. There is compelling evidence that childhood leukemia originates early in life with initial genetic alterations occurring in utero or before conception [1, 14].

In view of a possible early origin of childhood leukemia, many studies have investigated associations between perinatal characteristics and later development of disease. A number of studies found decreased risks of acute lymphoblastic leukemia (ALL) for children with older siblings, supporting the hypothesis that increased exposure to infections may be protective [15–19]. However, several large cohort studies found no evidence of such an effect [20–23]. Any protective effect of high birth order might be mediated by the birth interval between a newborn and older siblings yet only few studies have accounted for this with mixed results [22, 24, 25]. Higher parental age at birth was also found to be associated with the risk of both ALL and acute myeloid leukemia (AML) in recent analyses of pooled datasets and meta-analyses [26–28]. High birth weight is an established risk factor for childhood leukemia and ALL [29–32]. Recent studies have consistently shown positive associations between high birth weight relative to gestational age [33]. Finally, some early studies suggested that multiple birth may protect against later development of childhood leukemia [34, 35]. However, these findings could not be reproduced in recent studies that consistently adjusted for birth weight [20, 36].

We aimed to investigate the associations between childhood leukemia and perinatal characteristics in a nationwide register-based case–control study in Switzerland. We hypothesized that leukemia risk would be positively associated with higher maternal and paternal age, and negatively associated with higher birth order and a shorter birth interval to the next older sibling. In contrast to an earlier Swiss study of birth characteristics and childhood leukemia, which was based on partially overlapping data [19], this study is based on a larger sample size and considers a broader range of perinatal data.

## Materials and methods

### Population

We identified all primary diagnoses of leukemia registered in the Swiss Childhood Cancer Registry (SCCR) between 1981 and 2015 in children who were aged 0–15 years at diagnosis and had been born in Switzerland between 1969 and 2015. The SCCR is a nation-wide population-based cancer registry recording cancers in children and adolescents since 1976. It includes an estimated 91% of all diagnoses since the late 1980s, and an estimated 95% since the mid-1990s [37]. We used probabilistic linkage to match SCCR cases with corresponding birth records from the vital statistics of the Swiss Federal Statistical Office (FSO). The linkage was based on first names, date of birth, sex, municipality of residence at birth, and date of birth of both parents and was performed with G-link 2.3 (Statistics Canada, Ottawa, Ontario).

We randomly sampled five controls (birth records not linked to a cancer case) per case without replacement, matched on date of birth (± 6 months), sex, and municipality of residence of the mother at birth. We matched on municipality to reduce potential confounding by spatially varying risk factors. In some small municipalities, the number of eligible controls was smaller than five, in which case we selected controls from adjacent municipalities (first-order neighbors). This occurred for 3.0% of all controls.

### Outcomes and perinatal characteristics

We separately investigated the following outcomes based on the International Classification of Childhood Cancers, Third Edition (ICCC-3) [38]: leukemia (ICCC-3 main group-I), lymphoid leukemia (I.a) and acute myeloid leukemias (AML, I.b). Because chronic lymphocytic leukemia (CLL) (included in I.a) is exceedingly rare in children, we refer to ICCC-3 subgroup I.a as ALL. There were no cases of CLL in our study sample. We ran separate analyses for children aged 0–15 years, and 0–4 years for leukemia given the characteristic peak incidence among children under 5 years of age.

We investigated associations with the following perinatal characteristics: birth order in the sequence of live births by the same mother (first born, second, third, or fourth or later born), birth interval to the next older sibling (first born, 1–2 years, 3–4 years, or 5+ years), age of the mother and of the father at date of birth (< 25, 25–29, 30–34, 35–39, 40+ years), birth weight (< 2,500, 2,500–3,999, ≥ 4,000 g), multiple birth (singleton or multiple birth), and nationality of the mother (Swiss or other). We calculated pairwise Spearman’s rank correlations between the included birth characteristics.

We extracted all data on perinatal characteristics for both cases and controls from their birth records. In Swiss birth records, birth order and date of birth of the last previous birth are recorded only for births by married mothers, which accounted for 90.7% of total births between 1969 and 2015. Parental age and birth interval to the next older sibling were calculated as completed life years at the index child’s date of birth. Paternal age and the date of birth of the last previous birth were recorded only since 1979, and between 1979 and 1986 only year of birth instead of date of birth was recorded for mothers and fathers. For these years, we randomly attributed a specific date of the year of birth to calculate parental age. For multiple births, birth order was recorded in the order of actual births, i.e., in twins the later born is one rank higher than the earlier born. The birth interval to the next older sibling though was set to be the same for every child of a multiple birth and calculated as for a singleton birth, i.e., the number of years since the last previous birth if a woman had already given birth before and 0 otherwise.

### Statistical analysis

We used conditional logistic regression to investigate associations between leukemia, ALL, and AML and perinatal characteristics conditioning on the matched case–control sets. We fitted univariable models for every perinatal characteristic separately and multivariable models including all characteristics simultaneously to obtain crude and adjusted odds-ratios (OR) and 95% confidence intervals (CI). Likelihood ratio tests were performed to compare models with and without a given perinatal characteristic.

For leukemia and ALL we tested for possible interactions between pairs of perinatal characteristics that have recently been reported in the literature using likelihood ratio tests. Specifically, we tested for interactions between birth order (dichotomized first-born vs. later-born) and age of the father as well as between birth order and birth weight [39]. We also ran separate conditional logistic regression models including birth weight as a continuous variable and as a 7-level categorical variable (septiles) to investigate a possible U-shape of the association for AML [29, 31].

In sensitivity analyses, we (i) included only one (randomly sampled) twin from every twin pair of children with concordant leukemia; and (ii) excluded case–control sets of cases with trisomy 21.

All statistical analyses were performed using the R language for statistical computing version 3.6.0 [40] and STATA 15.1 (StataCorp, College Station, TX, USA).

## Results

We identified 1,623 children diagnosed with leukemia at age 0–15 years during 1981–2015 and born in Switzerland between 1969 and 2015. Of these, 225 (14%) could not be linked with a birth record, leaving 1,403 cases for analysis. Among the included cases, 1,144 (82%) were diagnosed with ALL and 179 (13%) with AML (Table 1). Of all leukemia cases, 576 (41%) were first-born, whereas 57 (4.1%) were fourth or later births by the same mother (Table S1 in the online supplementary material). Among cases, the prevalence of high birth weight (≥ 4000 g, 10.5% vs. 8.2%) and multiple birth (3.5% vs. 2.2%) was higher than among controls; 49 cases were born as part of a twin pair (Table S1). We found strong positive correlations between birth order and interval to next older sibling and between maternal and paternal age, and a negative correlation between multiple birth and birth weight (Fig. S1).

**Table 1:** Included cases of childhood leukemia

Characteristic	1,403	100.0%
ALL	1,144	81.5%
AML	179	12.8%
Sex		
Girls	581	41.4%
Boys	822	58.6%
Age at diagnosis		
< 5 years	762	54.3%
5+ years	641	45.7%
Year of diagnosis		
1981–1985	63	4.5%
1986–1990	123	8.8%
1991–1995	197	14.0%
1996–2000	223	15.9%
2001–2005	253	18.0%
2006–2010	238	17.0%
2011–2015	306	21.8%
Twins	49	3.5%
Trisomy 21	43	3.1%

We observed an increased risk of childhood leukemia at age 0–15 years among children with high birth weight compared to normal weight (adjusted odds ratio [OR] 1.37, 95% confidence interval [CI] 1.12–1.69) (Table 2). The adjusted model also indicated a reduced risk for low birth weight (adjusted OR 0.77, CI 0.56–1.05) (Table 2). We also found evidence of an increased risk of developing leukemia among children from multiple births compared to singletons which became stronger after adjusting for other covariates (adjusted OR 1.89, CI 1.24–2.86). Although there was no evidence for an association with maternal age (*p* value from LR test 0.71), the estimated risk was lowest for the youngest age category (< 25 years, reference) (Table 2). By contrast, we found no evidence of an association with other perinatal characteristics (*p* values of LR tests all > 0.4; Table 2).

**Table 2:** Associations between perinatal characteristics and childhood leukemia, diagnosed at age 0–15 years

Exposure	Categories	Cases	Crude OR^a^	95% CI^b^	*p* value^c^	Cases	Adjusted OR^d^	95% CI^b^	*p* value^c^
Birth order	First born	576	1.00		0.91	554	1.00		0.98
	Second	474	1.00	(0.88–1.14)		458	1.09	(0.57–2.10)	
	Third	172	1.06	(0.88–1.28)		166	1.12	(0.57–2.22)	
	Fourth or later	57	1.07	(0.79–1.46)		55	1.13	(0.54–2.34)	
Interval to next older sibling	First born	570	1.00		0.52	569	1.00		0.57
	1–2 years	400	1.00	(0.86–1.15)		398	0.89	(0.46–1.72)	
	3–4 years	159	0.96	(0.80–1.17)		159	0.87	(0.44–1.70)	
	5+ years	108	1.18	(0.93–1.48)		107	1.05	(0.52–2.09)	
Age of mother	< 25 years	228	1.00		0.65	188	1.00		0.71
	25–29 years	486	1.08	(0.91–1.28)		427	1.10	(0.89–1.35)	
	30–34 years	467	1.13	(0.95–1.35)		426	1.19	(0.94–1.51)	
	35–39 years	186	1.09	(0.88–1.36)		165	1.16	(0.87–1.56)	
	40+ years	36	1.25	(0.84–1.85)		27	1.16	(0.70–1.91)	
Age of father	< 25 years	80	1.00		0.97	80	1.00		0.66
	25–29 years	305	0.93	(0.71–1.22)		303	0.86	(0.64–1.15)	
	30–34 years	448	0.93	(0.71–1.21)		440	0.79	(0.58–1.08)	
	35–39 years	281	0.95	(0.72–1.25)		273	0.79	(0.56–1.09)	
	40+ years	140	0.98	(0.71–1.34)		137	0.81	(0.56–1.17)	
Birth weight	< 2,500 g	71	0.94	(0.73–1.23)	0.022	60	0.77	(0.56–1.05)	0.002
	2,500–3,999 g	1,141	1.00			1,034	1.00		
	≥ 4,000 g	148	1.31	(1.08–1.59)		139	1.37	(1.12–1.69)	
Multiple birth	Singleton	1,354	1.00		0.004	1,189	1.00		0.004
	Multiple	49	1.68	(1.20–2.36)		44	1.89	(1.24–2.86)	
Nationality of mother	Swiss	1,039	1.00		0.23	911	1.00		0.41
	Other	361	0.92	(0.80–1.06)		322	0.94	(0.80–1.10)	

^a^Odds ratio of raw conditional logistic regression models

^b^95% confidence interval

^c^*p* value of likelihood ratio test comparing model with and without a given perinatal characteristic

^d^Odds ratio of conditional logistic regression models adjusting for birth order, interval to next older sibling, age of mother, age of father, birth weight, multiple birth, and nationality of mother

Results for ALL at 0–15 years (Table 3) and leukemia under 5 years of age (Table 4) were broadly similar. Strong evidence for an increased risk of ALL at 0–15 years was again observed in the adjusted models for high birth weight (adjusted OR 1.50, CI 1.19–1.87) and being part of a multiple birth (adjusted OR 1.82, CI 1.15–2.88) (Table 3). The association with multiple birth was stronger for leukemia diagnosed under 5 years of age (adjusted OR 2.23, CI 1.32–3.76) than for ALL at 0–15 years (Table 4).

**Table 3:** Associations between perinatal characteristics and ALL, diagnosed at age 0–15 years

Exposure	Categories	Cases	Crude OR^a^	95% CI^b^	*p* value^c^	Cases	Adjusted OR^d^	95% CI^b^	*p* value^c^
Birth order	First born	468	1.00		0.73	448	1.00		0.67
	Second	397	1.07	(0.93–1.24)		383	1.12	(0.53–2.36)	
	Third	135	1.01	(0.82–1.25)		130	1.02	(0.47–2.21)	
	Fourth or later	43	0.92	(0.65–1.31)		41	0.93	(0.40–2.13)	
Interval to next older sibling	First born	461	1.00		0.71	460	1.00		0.72
	1–2 years	331	1.03	(0.88–1.21)		329	0.92	(0.44–1.95)	
	3–4 years	128	1.00	(0.80–1.24)		128	0.90	(0.42–1.92)	
	5+ years	86	1.16	(0.90–1.50)		85	1.07	(0.49–2.34)	
Age of mother	< 25 years	182	1.00		0.37	147	1.00		0.24
	25–29 years	393	1.10	(0.91–1.34)		345	1.15	(0.90–1.45)	
	30–34 years	386	1.21	(1.00–1.48)		356	1.34	(1.03–1.75)	
	35–39 years	155	1.11	(0.87–1.41)		135	1.25	(0.90–1.73)	
	40+ years	28	1.25	(0.80–1.95)		19	1.08	(0.61–1.94)	
Age of father	< 25 years	65	1.00		0.95	65	1.00		0.68
	25–29 years	241	0.93	(0.68–1.26)		239	0.84	(0.61–1.16)	
	30–34 years	373	0.99	(0.74–1.33)		371	0.81	(0.58–1.14)	
	35–39 years	229	0.96	(0.71–1.31)		222	0.76	(0.53–1.09)	
	40+ years	107	0.93	(0.66–1.32)		105	0.76	(0.50–1.16)	
Birth weight	< 2,500 g	57	0.95	(0.71–1.27)	0.006	50	0.83	(0.59–1.17)	0.001
	2,500–3,999 g	923	1.00			833	1.00		
	≥ 4,000 g	126	1.42	(1.15–1.75)		119	1.50	(1.19–1.87)	
Multiple birth	Singleton	1,105	1.00		0.027	966	1.00		0.013
	Multiple	39	1.55	(1.07–2.25)		36	1.82	(1.15–2.88)	
Nationality of mother	Swiss	850	1.00		0.34	745	1.00		0.60
	Other	291	0.93	(0.79–1.08)		257	0.95	(0.80–1.14)	

^a^Odds ratio of raw conditional logistic regression models

^b^95% confidence interval

^c^*p* value of likelihood ratio test comparing model with and without a given perinatal characteristic

^d^Odds ratio of conditional logistic regression models adjusting for birth order, interval to next older sibling, age of mother, age of father, birth weight, multiple birth, and nationality of mother

**Table 4:** Associations between perinatal characteristics and childhood leukemia, diagnosed at age 0–4 years

Exposure	Categories	Cases	Crude OR^a^	95% CI^b^	*p* value^c^	Cases	Adjusted OR^d^	95% CI^b^	*p* value^c^
Birth order	First born	327	1.00		0.32	325	1.00		0.71
	Second	241	0.87	(0.73–1.04)		239	0.94	(0.40–2.22)	
	Third	88	0.94	(0.72–1.22)		87	0.95	(0.38–2.35)	
	Fourth or later	25	0.74	(0.47–1.15)		25	0.72	(0.27–1.92)	
Interval to next older sibling	First born	335	1.00		0.23	334	1.00		0.81
	1–2 years	206	0.83	(0.68–1.00)		205	0.87	(0.37–2.05)	
	3–4 years	83	0.86	(0.66–1.12)		83	0.91	(0.38–2.21)	
	5+ years	54	0.99	(0.71–1.36)		54	1.02	(0.41–2.56)	
Age of mother	< 25 years	112	1.00		0.80	95	1.00		0.32
	25–29 years	252	1.01	(0.79–1.29)		227	1.11	(0.83–1.49)	
	30–34 years	260	1.12	(0.87–1.43)		237	1.34	(0.96–1.86)	
	35–39 years	115	1.10	(0.82–1.48)		101	1.41	(0.94–2.10)	
	40+ years	23	1.19	(0.72–1.96)		16	1.12	(0.57–2.17)	
Age of father	< 25 years	38	1.00		0.94	38	1.00		0.75
	25–29 years	162	1.01	(0.68–1.49)		160	0.94	(0.62–1.43)	
	30–34 years	253	1.03	(0.70–1.50)		246	0.89	(0.57–1.38)	
	35–39 years	156	0.96	(0.64–1.42)		150	0.79	(0.49–1.26)	
	40+ years	85	1.08	(0.70–1.68)		82	0.90	(0.53–1.52)	
Birth weight	< 2,500 g	49	1.20	(0.86–1.65)	0.10	40	0.95	(0.65–1.39)	0.032
	2,500–3,999 g	631	1.00			562	1.00		
	≥ 4,000 g	78	1.30	(1.00–1.70)		74	1.47	(1.11–1.95)	
Multiple birth	Singleton	728	1.00		< 0.001	647	1.00		0.004
	Multiple	34	2.21	(1.45–3.37)		29	2.23	(1.32–3.76)	
Nationality of mother	Swiss	552	1.00		0.60	488	1.00		0.81
	Other	209	0.95	(0.79–1.15)		188	1.03	(0.83–1.26)	

^a^Odds ratio of raw conditional logistic regression models

^b^95% confidence interval

^c^*p* value of likelihood ratio test comparing model with and without a given perinatal characteristic

^d^Odds ratio of conditional logistic regression models adjusting for birth order, interval to next older sibling, age of mother, age of father, birth weight, multiple birth, and nationality of mother

Results for AML at 0–15 years of age were rather different except for multiple birth, which was also associated with an increased risk although the evidence was weaker (adjusted OR 2.44, CI 0.55–10.77) (Table 5). There was evidence of an increased risk associated with higher birth order (*p* LR test = 0.04 in the adjusted model) (Table 5). Adjusted ORs were 1.76 (CI 0.25–12.22) and 3.08 (CI 0.43–22.03) in third-born and fourth- or later-born children, respectively. By contrast, there was little evidence of an association for the other birth characteristics (Table 5).

**Table 5:** Associations between perinatal characteristics and AML, diagnosed at age 0–15 years

Exposure	Categories	Cases	Crude OR^a^	95% CI^b^	*p* value^c^	Cases	Adjusted OR^d^	95% CI^b^	*p* value^c^
Birth order	First born	72	1.00		0.006	71	1.00		0.037
	Second	53	0.72	(0.49–1.05)		52	0.95	(0.15–6.01)	
	Third	29	1.39	(0.84–2.30)		28	1.76	(0.25–12.22)	
	Fourth or later	10	2.73	(1.18–6.32)		10	3.08	(0.43–22.03)	
Interval to next older sibling	First born	73	1.00		0.52	73	1.00		0.74
	1–2 years	47	0.83	(0.55–1.24)		47	0.75	(0.12–4.70)	
	3–4 years	24	0.93	(0.56–1.55)		24	0.78	(0.12–5.10)	
	5+ years	17	1.33	(0.71–2.51)		17	1.11	(0.16–7.65)	
Age of mother	< 25 years	25	1.00		0.27	22	1.00		0.15
	25–29 years	74	1.46	(0.89–2.40)		66	1.80	(0.95–3.42)	
	30–34 years	54	0.99	(0.59–1.67)		48	1.20	(0.59–2.46)	
	35–39 years	20	1.31	(0.67–2.55)		19	1.37	(0.56–3.39)	
	40+ years	6	1.74	(0.61–5.01)		6	2.90	(0.72–11.64)	
Age of father	< 25 years	10	1.00		0.13	10	1.00		0.17
	25–29 years	47	1.04	(0.48–2.22)		47	0.73	(0.31–1.76)	
	30–34 years	53	0.68	(0.32–1.44)		51	0.45	(0.18–1.12)	
	35–39 years	31	0.76	(0.34–1.71)		30	0.48	(0.17–1.31)	
	40+ years	23	1.30	(0.55–3.12)		23	0.68	(0.23–2.03)	
Birth weight	< 2,500 g	7	0.64	(0.29–1.46)	0.29	6	0.41	(0.14–1.25)	0.18
	2,500–3,999 g	156	1.00			143	1.00		
	≥ 4,000 g	13	0.69	(0.37–1.29)		12	0.76	(0.39–1.51)	
Multiple birth	Singleton	174	1.00		0.39	156	1.00		0.24
	Multiple	5	1.61	(0.57–4.60)		5	2.44	(0.55–10.77)	
Nationality of mother	Swiss	128	1.00		0.77	113	1.00		0.97
	Other	51	1.06	(0.73–1.54)		48	1.01	(0.66–1.55)	

^a^Odds ratio of raw conditional logistic regression models

^b^95% confidence interval

^c^*p* value of likelihood ratio test comparing model with and without a given perinatal characteristic

^d^Odds ratio of conditional logistic regression models adjusting for birth order, interval to next older sibling, age of mother, age of father, birth weight, multiple birth, and nationality of mother

Testing for interactions between perinatal characteristics, we found weak evidence of an interaction between birth order (dichotomized first-born vs. later-born) and birth weight in the adjusted models for ALL diagnosed at 0–15 years (*p* LR test = 0.049) and leukemia under 5 years of age (*p* LR test = 0.073) but not for leukemia overall (*p* LR test = 0.20) (Tables S3–S5). Estimated interaction terms indicted that, among children with high or low birth weight, those born second or later had lower risks compared to first-borns (Tables S3–S5). By contrast, we found no evidence of an interaction between birth order and age of the father for any of the three outcomes (*p* LR tests all > 0.5; results not shown).

**Fig. 1:**
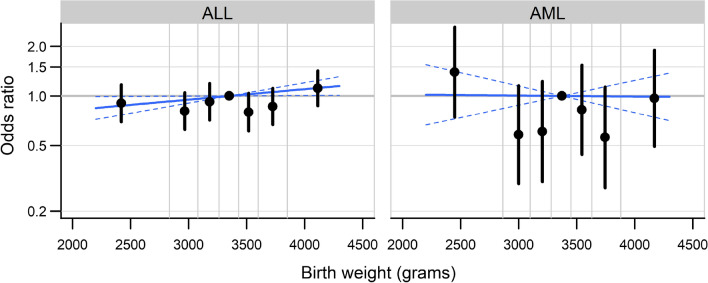
Association between birth weight and ALL and AML, diagnosed at age 0–15 years. Adjusted odds ratios (black dots) and 95% confidence intervals for septiles of birth weight compared to the mid-septile (the points are plotted against mean birth weight within septiles); Odds ratio (blue line) of birth weight as a continuous variable in an otherwise identical conditional logistic regression model. (Color figure online)

Assessing the functional shape of the association with birth weight, the adjusted model using septiles of birth weight as a categorical variable showed no indication of a monotonous increase in ALL risk (*p* LR test = 0.13; Fig. 1). A positive linear association was observed though when including birth weight as a continuous variable with an estimated adjusted OR per 1000 g positive difference in birth weight of 1.16 (CI 1.01–1.33). This trend seems to be driven by children with high birth weights (highest septile) (Fig. 1). For AML, the adjusted model using septiles of birth weight showed weak evidence on an association (*p* LR test = 0.06; Fig. 1), however, there was no indication of a linear trend for birth weight as a continuous variable (adjusted OR 0.99, 0.69–1.41).

In sensitivity analyses, we included only one twin from each of the four twin pairs with concordant diagnoses of leukemia we identified in the SCCR. In this analysis, the odds ratio for being part of a multiple birth was slightly reduced compared to the main analysis (adjusted OR 1.80, 1.18–2.75) but the other results remained similar (Table S6). Excluding cases with trisomy 21 (*n* = 43) reduced the estimated OR of leukemia for mothers of the age group ≥ 40 years (adjusted OR 0.85, 0.49–1.48) but left other estimates unchanged (Table S7). Following the suggestion of a reviewer, we repeated the main analyses excluding case–control sets of cases from multiple births. Results from these analyses were very similar to the main analysis (Tables S8–S11).

## Discussion

### Main findings

This register-based case–control study showed strong evidence of an increased risk of leukemia among children with high birth weight (≥ 4,000 g) and children from multiple births. These associations were similar for ALL and stronger for children diagnosed under the age of 5 years. For AML, there was little evidence for associations with birth weight and multiple births, although including birth weight as a 7-level categorical variable (septiles) was suggestive of a non-linear association, which by visual inspection is compatible with a U-shape (Fig. 1). However, we did find evidence of an increased risk of AML in third or later-born children compared to first-born children. There was little evidence of an association for the other investigated birth characteristics, although estimated ORs for leukemia and ALL in the offspring of mothers belonging to older age groups were consistently higher than in those of mothers aged less than 25 years.

### Results in the context of previous studies

Our findings are compatible with the results of our previous study [19] using partially overlapping data from the SCCR. That study found an increased risk of ALL for higher age of the mother and a weak protective effect of having older siblings in the same household in children under 5 years of age but not in older children. Compared to the present study, that study was based on a smaller sample comprising 425 children with ALL and did not include data on multiple births and birth weight.

Our current findings on birth weight concur with the literature that has established high birth weight as a risk factor for several childhood cancers including leukemia. Several pooled and meta-analyses have observed a linear trend between birth weight and childhood leukemia or ALL, or both [29–32, 41]. Our model estimate for ALL for birth weight as a continuous linear term (adjusted OR 1.16/1,000-g increase in birth weight) matched these findings closely (pooled OR 1.18, CI 1.13–1.23 [29]; pooled OR 1.14, CI 1.08–1.20 [30]). Accelerated fetal growth might serve as explanation as suggested by a recent pooled analysis by the Childhood Leukemia International Consortium (CLIC) showing a strong consistent positive association with increased risk of ALL [33]. However, we lacked information on gestational age (which has been collected in Swiss birth records only since 2006) and could thus not account for this. By contrast, the evidence on the association between birth weight and childhood AML is more heterogeneous with both pooled and meta-analyses suggesting a U-shaped association [17, 29, 31]. However, a recent CLIC study found a positive association for AML with high birth weight and with being large-for-gestational-age but no indication of a U-shaped association [42].

Regarding the association between parental age and ALL, our findings are compatible with the results of a meta-analysis of 77 studies [26] and a recent CLIC study [27] showing a monotonically increasing risk with higher age of the mother that was stronger among children under 5 years of age. The same study also observed an increased risk of ALL for higher age of the father. However, two recent large register-based studies in Sweden and California [20, 43], like our study, found no evidence of such an association. For AML, our findings were suggestive of a lower risk among children of young mothers but a higher risk among children of young fathers, though the evidence was weak. This is compatible with findings of the meta-analysis which observed an increased risk of AML in the offspring of the youngest fathers only [26]. However, a recent pooled analysis by CLIC observed no association between paternal age and risk of AML [28].

Higher birth order was found to be associated with reduced risk of ALL in a number of studies [16–18, 24]. However, results from cohort studies were mostly inconclusive [20–23]. A meta-analysis by the CLIC consortium reported a slight protective effect of higher birth order but with significant heterogeneity between individual studies [44]. More recently, the International Childhood Cancer Cohort Consortium (I4C) reported a protective effect for higher birth order that was based on only 185 cases of ALL [39]. For AML, our study results are consistent with a number of studies including a review reporting some evidence of increased risks for higher birth order [45]. Only few studies also looked at the actual birth interval between siblings. Two studies reported increased risks for ALL associated with birth intervals larger than 3 and 5 years, respectively [22, 46], while other studies, like ours, found no evidence of an association [25, 47] or a protective effect for birth intervals of less than 5 years [24]. None of these studies reported evidence of any association for AML. Our results also support a recent report that the effect of birth order on risk of ALL might be modified by birth weight [39]. Specifically, for children with low birth weight (< 2,500 g), the risk of ALL under 5 years was reduced in our study among later-born compared to first-born children.

This is the first study to report evidence of an increased risk of childhood leukemia and ALL in children from multiple births. While a number of studies of perinatal characteristics have included multiple birth as a covariate in multivariate models, no large cohort or case–control study has reported evidence of any association with childhood leukemia [20, 25, 48]. Studies specifically comparing twins with singletons have reported either a reduced leukemia risk for same sex twins [34] or found no evidence of an effect on leukemia risk [36].

### Strengths and weaknesses

Because we identified cases from a population-based cancer registry of high coverage and sampled controls from national birth records, the risk of selection bias in our study is limited. Information on birth characteristics was obtained from the same source, namely the birth records, for cases and controls at the time of birth, suggesting that our study is free of recall bias or differential misclassification. We were able to include leukemia cases diagnosed during a period of over three decades, allowing us to achieve a sample size that is comparable with the most recent European case–control or cohort studies. Matching by date of birth and municipality of residence of the mother at time of birth should have reduced the potential risk of confounding by spatial or temporal variation in environmental risk factors, such as traffic-related air-pollution [6, 49], background ionizing radiation [5, 50], or agricultural pesticides [10, 11].

Probabilistic record linkage of cases with their birth records may have resulted in some misclassifications of the outcomes. However, this risk appears small as the outcomes considered are rare and the linkage led to non-ambiguous matches in the overwhelming majority of cases. A considerable proportion of children had missing data for birth order (10%), birth interval to the next older sibling (13%), and age of the father (11%) and excluding these children may have introduced some selection bias if data were not missing at random [51]. This risk may be compounded by the fact that in the birth records, due to administrative procedures of record keeping, parity of the mother is recorded only for married but not for single or divorced women. However, the number of births to unmarried parents made up less than 10% of all births in the national birth registry and rates of missingness varied only minimally between cases of the different outcomes and controls (Table S2). Moreover, we did not require controls to be living in Switzerland at the time of the cases’ diagnosis (a requirement for cases), which may have introduced selection bias. The direction of this possible bias is unclear though. Finally, in an observational study, we cannot exclude residual confounding by risk factors we did not have information on. In particular, we had no information on certain perinatal characteristics of interest such as gestational age [33], mode of delivery [52], or use of artificial reproduction technologies [53].

### Interpretation

It is unclear what might explain the observed association between multiple birth and childhood leukemia in our study. Newborns from multiple births typically have lower birth weight. Early studies reporting a reduced risk were often small and did not (consistently) control for birth weight (reviewed in [35]). Another study of any childhood cancers (all diagnostic groups, not only leukemias) in twins that partially controlled for birth weight found a reduced overall risk but noted an excess of cases among twins with birth weight of 3 kg and higher [35]. In our study, we adjusted for birth weight, and low birth weight was associated with a decreased risk of leukemia, and so cannot explain an increased risk among children from multiple births. Moreover, it is known, that if one twin has been diagnosed with childhood leukemia, the other has an increased risk of also developing the disease, allegedly due to the intra-placental transfer of pre-leukemic clones from one fetus to the other [1, 54]. However, this could not account for the observed effect either as the sensitivity analysis in which we excluded one twin from each of the concordant pairs has shown. This analysis simulates a situation, in which the in utero exposure to a pre-leukemic clone has no effect because the second twin, by being excluded from the analysis, is considered to have been unaffected by leukemia. If the in utero transfer of a pre-leukemic clone was the main causal pathway underlying the increased leukemia risk of twins, the risk among twins in this analysis should resemble that of singletons. Another possible explanation might be an adverse event of medical interventions associated with multiple birth such as fertility treatments, medically assisted reproduction (MAR) [53, 55], and prelabour cesarean delivery [52]. In the FSO birth records, the proportion of multiple births among total births increased almost linearly with maternal age, from 1.6% among mothers aged 25 years or younger to 4.2% among those aged 40 years and above. We lacked data on individual use of MAR but, according to the FSO, the number of children conceived by MAR has risen from 910 in 2002 to 2,020 in 2015, making up 2.3% of total live births that year. Moreover, the proportion of multiple births among all children conceived by MAR has fluctuated between 15.5% and 22.8% over this period. In order to address the possibility that multiple birth may be associated with leukemia because in recent years it has become almost a marker for MAR, we carried out an ex post analysis subsetting our data by decade (1980–1989, 1990–1999, 2000–2009). We found that compared to the 1980s, the risk of CL associated with multiple birth was increased for children born during the 1990s when MAR was gaining ground but not during the 2000s when it became even more common (Tables S12–S14), therefore providing no clear support for this hypothesis. If indeed such interventions explained the observed association in our study, the question arises, why recent large studies found no evidence of an increased risk among children of multiple births [16, 43], although some studies reported odds ratios above 1.2 for leukemias [20] or even higher in some age subgroups [36].

Regarding other perinatal characteristics our investigation was either compatible (maternal age) with or provided further support (birth weight) for expected associations [27, 33]. Moreover, our study confirmed a recent report of an interaction adding an enhanced protective effect to later born children with low birth weight [39] that, due to the lack of data on gestational age and limited sample sizes, we could not explore fully but merits further investigation in future studies. A possible explanation offered by the authors of that study is that the compounded effect of the greater risk of infections that low birth weight babies have in early life together with the increased early exposure to infections that comes from having older siblings could result in a stronger immune response.

## Conclusions

Our results add to the existing evidence that high birth weight is associated with an increased risk of childhood leukemia. Furthermore, our study is the first to report evidence suggesting that children from multiple births are at increased risk for developing leukemia overall and ALL specifically.

## Supplementary Information

Below is the link to the electronic supplementary material.

Supplementary file1 (PDF 282 kb)

## Data Availability

The Swiss Childhood Cancer Registry is the permanent repository of data on childhood cancer cases used in this study. This data cannot be made publicly available for both legal and ethical reasons as this would compromise patient confidentiality and participant privacy. Interested researchers may contact the corresponding author or the Swiss Childhood Cancer Registry (http://childhoodcancerregistry.ch/) via its online contact form for further information.
