# Supplementary material for: Birth characteristics and childhood leukemia in Switzerland: a register-based case–control study
Source: Cancer Causes Control. 2021 Apr 20;32(7):713–23. doi: 10.1007/s10552-021-01423-3 (PMC8184536; doi:10.1007/s10552-021-01423-3)

Supplementary Material

Birth characteristics and childhood leukemia in Switzerland: a register-based case-control study

Judith E. Lupatsch[1,2‡], Christian Kreis[1‡], Garyfallos Konstantinoudis[1,3], Marc Ansari[4,5], Claudia E. Kuehni[1], Ben D. Spycher[1*]

1 Institute of Social and Preventive Medicine, University of Bern, Bern, Switzerland

2 Institute of Pharmaceutical Medicine, University of Basel, Basel, Switzerland

3 MRC Centre of Environment and Health, Department of Epidemiology and Biostatistics, School of Public Health, Imperial College London, London, United Kingdom

4 CANSEARCH Research Laboratory, Department of Paediatrics, Gynaecology and Obstetrics, Geneva, University, Geneva, Switzerland

5 Onco-Hematology Unit, Department of Women, Child and Adolescent, Geneva University Hospital, Geneva, Switzerland

[‡]Contributed equally

Corresponding Author

*Ben D. Spycher

Institute of Social and Preventive Medicine (ISPM), University of Bern, Mittelstrasse 43, 3012 Bern, Switzerland.

Email: ben.spycher@ispm.unibe.ch

Phone: +41 31 631 33 46

Tables

Table S1. Perinatal characteristics of cases and controls

Exposure	Categories	Cases	%	Controls	%	p-value[a]
Birth order	1st born	576	41.1%	2,875	41.0%	0.890
	2nd	474	33.8%	2,371	33.8%	
	3rd	172	12.3%	809	11.5%	
	4th or later	57	4.1%	265	3.8%	
	missing[b]	124	8.8%	695	9.9%	
Interval to next older sibling	1st born	570	40.6%	2,841	40.5%	0.890
	1-2 y	400	28.5%	1,982	28.3%	
	3-4 y	159	11.3%	838	11.9%	
	5+ y	108	7.7%	452	6.4%	
	missing[c]	166	11.8%	902	12.9%	
Age of mother	<25 y	228	16.3%	1,236	17.6%	0.391
	25-29 y	486	34.6%	2,441	34.8%	
	30-34 y	467	33.3%	2,246	32.0%	
	35-39 y	186	13.3%	929	13.2%	
	40+ y	36	2.6%	158	2.3%	
	missing	0	0.0%	5	0.1%	
Age of father	<25 y	80	5.7%	375	5.3%	0.906
	25-29 y	305	21.7%	1,544	22.0%	
	30-34 y	448	31.9%	2,261	32.2%	
	35-39 y	281	20.0%	1,372	19.6%	
	40+ y	140	10.0%	648	9.2%	
	missing[d]	149	10.6%	815	11.6%	
Birth weight	<2500 g	71	5.1%	383	5.5%	0.018
	2500-3999 g	1,141	81.3%	5,818	82.9%	
	≧4000 g	148	10.5%	577	8.2%	
	missing	43	3.1%	237	3.4%	
Multiple birth	singleton	1,354	96.5%	6,859	97.8%	0.003
	multiple birth	49	3.5%	151	2.2%	
Nationality of mother	Swiss	1,039	74.1%	5,083	72.5%	0.267
	other	361	25.7%	1,902	27.1%	
	missing	3	0.2%	30	0.4%	

[a]P-value of X^2-tests; [b]Birth order is recorded only for births by married mothers; [c]Date of the last previous birth has been recorded since 1979 but only for births by married mothers; [d]Paternal age has been recorded since 1979.

Table S2. Marital status of mothers of cases and controls

Outcome	Single	Married	Divorced	Widowed	Missing	Total
Leukemia	7.20%	91.23%	1.43%	0.14%	0.00%	1,403
ALL	7.34%	91.17%	1.40%	0.09%	0.00%	1,144
AML	6.15%	92.18%	1.12%	0.56%	0.00%	179
Controls	8.11%	90.11%	1.61%	0.10%	0.07%	7,015

Table S3. Associations between perinatal characteristics and childhood leukemia, diagnosed at age 0-15 years, including an interaction term between birth order (dichotomized first-born vs. later-born) and birth weight. P-value of LR test (comparing conditional logistic regression model with and without interaction term) = 0.20

Exposure	Categories	Cases	Adjusted OR[a]	95% CI[b]
Birth order	1st born	554	1.00	
	2nd or later	679	1.23	(0.62-2.41)
Interval to next older sibling	1st born	569	1.00	
	1-2 y	398	0.84	(0.43-1.63)
	3-4 y	159	0.82	(0.41-1.62)
	5+ y	107	0.99	(0.49-1.99)
Age of mother	<25 y	188	1.00	
	25-29 y	427	1.10	(0.89-1.36)
	30-34 y	426	1.19	(0.94-1.51)
	35-39 y	165	1.17	(0.87-1.57)
	40+ y	27	1.17	(0.71-1.93)
Age of father	<25 y	80	1.00	
	25-29 y	303	0.85	(0.64-1.15)
	30-34 y	440	0.79	(0.58-1.08)
	35-39 y	273	0.78	(0.56-1.09)
	40+ y	137	0.80	(0.55-1.17)
Birth weight	<2500 g	60	0.88	(0.60-1.31)
	2500-3999 g	1,034	1.00	
	≧4000 g	139	1.67	(1.21-2.30)
Multiple birth	singleton	1,189	1.00	
	multiple	44	1.92	(1.26-2.91)
Nationality of mother	Swiss	911	1.00	
	other	322	0.94	(0.80-1.10)
2nd or later-born * <2500 g		27	0.73	(0.40-1.34)
2nd or later-born * ≧4000 g		81	0.72	(0.48-1.10)

[a]Odds ratio of conditional logistic regression models adjusting for birth order, interval to next older sibling, age of mother, age of father, birth weight, multiple birth, and nationality of mother; [b]95% confidence interval.

Table S4. Associations between perinatal characteristics and ALL, diagnosed at age 0-15 years, including an interaction term between birth order (dichotomized first-born vs. later-born) and birth weight. P-value of LR test (comparing conditional logistic regression model with and without interaction term) = 0.049

Exposure	Categories	Cases	Adjusted OR[a]	95% CI[b]
Birth order	1st born	448	1.00	
	2nd or later	554	1.40	(0.65-3.01)
Interval to next older sibling	1st born	460	1.00	
	1-2 y	329	0.77	(0.36-1.64)
	3-4 y	128	0.74	(0.34-1.60)
	5+ y	85	0.88	(0.40-1.93)
Age of mother	<25 y	147	1.00	
	25-29 y	345	1.14	(0.90-1.45)
	30-34 y	356	1.32	(1.01-1.73)
	35-39 y	135	1.23	(0.89-1.70)
	40+ y	19	1.08	(0.61-1.94)
Age of father	<25 y	65	1.00	
	25-29 y	239	0.83	(0.60-1.16)
	30-34 y	371	0.81	(0.57-1.14)
	35-39 y	222	0.74	(0.52-1.07)
	40+ y	105	0.74	(0.49-1.12)
Birth weight	<2500 g	50	1.11	(0.73-1.69)
	2500-3999 g	833	1.00	
	≧4000 g	119	1.84	(1.30-2.62)
Multiple birth	singleton	966	1.00	
	multiple	36	1.84	(1.17-2.90)
Nationality of mother	Swiss	745	1.00	
	other	257	0.96	(0.80-1.14)
2nd or later-born * <2500 g		19	0.50	(0.25-0.98)
2nd or later-born * ≧4000 g		70	0.70	(0.45-1.11)

[a]Odds ratio of conditional logistic regression models adjusting for birth order, interval to next older sibling, age of mother, age of father, birth weight, multiple birth, and nationality of mother; [b]95% confidence interval.

Table S5. Associations between perinatal characteristics and childhood leukemia, diagnosed at age 0-4 years, including an interaction term between birth order (dichotomized first-born vs. later-born) and birth weight. P-value of LR test (comparing conditional logistic regression model with and without interaction term) = 0.073

Exposure	Categories	Cases	Adjusted OR[a]	95% CI[b]
Birth order	1st born	325	1.00	
	2nd or later	351	1.16	(0.48-2.83)
Interval to next older sibling	1st born	334	1.00	
	1-2 y	205	0.75	(0.31-1.80)
	3-4 y	83	0.79	(0.32-1.94)
	5+ y	54	0.88	(0.35-2.22)
Age of mother	<25 y	95	1.00	
	25-29 y	227	1.12	(0.84-1.51)
	30-34 y	237	1.34	(0.97-1.87)
	35-39 y	101	1.42	(0.95-2.11)
	40+ y	16	1.12	(0.58-2.18)
Age of father	<25 y	38	1.00	
	25-29 y	160	0.92	(0.60-1.40)
	30-34 y	246	0.87	(0.56-1.35)
	35-39 y	150	0.76	(0.48-1.22)
	40+ y	82	0.85	(0.50-1.43)
Birth weight	<2500 g	40	1.17	(0.73-1.90)
	2500-3999 g	562	1.00	
	≧4000 g	74	2.02	(1.33-3.05)
Multiple birth	singleton	647	1.00	
	multiple	29	2.20	(1.31-3.70)
Nationality of mother	Swiss	488	1.00	
	other	188	1.04	(0.84-1.28)
2nd or later-born *	<2500 g	16	0.61	(0.28-1.32)
2nd or later-born *	≧4000 g	37	0.56	(0.32-0.99)

[a]Odds ratio of conditional logistic regression models adjusting for birth order, interval to next older sibling, age of mother, age of father, birth weight, multiple birth, and nationality of mother; [b]95% confidence interval.

Table S6. Associations between perinatal characteristics and childhood leukemia, diagnosed at age 0-15 years, excluding one twin from twin pairs with concordant diagnoses of leukemia

Exposure	Categories	Cases	Crude OR[a]	95% CI[b]	p-value[c]	Cases	Adjusted OR[d]	95% CI[b]	p-value[c]
Birth order	1st born	576	1.00		0.88	554	1.00		0.98
	2nd	471	0.99	(0.87-1.14)		455	0.98	(0.49-1.95)	
	3rd	172	1.06	(0.88-1.28)		166	1.02	(0.50-2.07)	
	4th or later	57	1.08	(0.79-1.46)		55	1.03	(0.48-2.19)	
Interval to next older sibling	1st born	568	1.00		0.50	567	1.00		0.57
	1-2 y	399	1.00	(0.86-1.15)		397	0.98	(0.50-1.96)	
	3-4 y	159	0.97	(0.80-1.18)		159	0.96	(0.48-1.94)	
	5+ y	108	1.18	(0.94-1.49)		107	1.16	(0.57-2.39)	
Age of mother	<25 y	228	1.00		0.66	188	1.00		0.71
	25-29 y	485	1.08	(0.91-1.28)		426	1.09	(0.88-1.35)	
	30-34 y	465	1.13	(0.95-1.35)		425	1.19	(0.94-1.51)	
	35-39 y	185	1.09	(0.88-1.36)		164	1.16	(0.87-1.56)	
	40+ y	36	1.25	(0.84-1.86)		27	1.16	(0.70-1.92)	
Age of father	<25 y	80	1.00		0.97	80	1.00		0.66
	25-29 y	305	0.93	(0.71-1.22)		303	0.86	(0.64-1.16)	
	30-34 y	446	0.92	(0.71-1.20)		439	0.80	(0.58-1.08)	
	35-39 y	281	0.95	(0.72-1.26)		273	0.79	(0.57-1.10)	
	40+ y	138	0.97	(0.71-1.33)		135	0.80	(0.55-1.16)	
Birth weight	<2500 g	67	0.89	(0.68-1.16)	0.015	57	0.75	(0.55-1.03)	0.002
	2500-3999 g	1,141	1.00			1,034	1.00		
	≧4000 g	148	1.31	(1.08-1.60)		139	1.37	(1.12-1.69)	
Multiple birth	singleton	1,354	1.00		0.019	1,189	1.00		0.008
	multiple	45	1.54	(1.09-2.17)		41	1.80	(1.18-2.75)	
Nationality of mother	Swiss	1,035	1.00		0.26	908	1.00		0.42
	other	361	0.92	(0.80-1.06)		322	0.94	(0.80-1.10)	

[a]Odds ratio of raw conditional logistic regression models; [b]95% confidence interval; [c]P-value of likelihood ratio test comparing model with and without a given perinatal characteristic; [d]Odds ratio of conditional logistic regression models adjusting for birth order, interval to next older sibling, age of mother, age of father, birth weight, multiple birth, and nationality of mother.

Table S7. Associations between perinatal characteristics and childhood leukemia, diagnosed at age 0-15 years, excluding cases with trisomy 21

Exposure	Categories	Cases	Crude OR[a]	95% CI[b]	p-value[c]	Cases	Adjusted OR[d]	95% CI[b]	p-value[c]
Birth order	1st born	560	1.00		0.95	538	1.00		0.97
	2nd	465	1.01	(0.89-1.16)		449	1.15	(0.59-2.22)	
	3rd	166	1.06	(0.87-1.29)		160	1.17	(0.59-2.32)	
	4th or later	52	1.01	(0.73-1.39)		50	1.12	(0.53-2.34)	
Interval to next older sibling	1st born	554	1.00		0.58	553	1.00		0.52
	1-2 y	393	1.01	(0.88-1.17)		391	0.87	(0.45-1.68)	
	3-4 y	151	0.95	(0.78-1.16)		151	0.82	(0.42-1.62)	
	5+ y	103	1.15	(0.91-1.46)		102	1.01	(0.50-2.03)	
Age of mother	<25 y	224	1.00		0.65	185	1.00		0.46
	25-29 y	475	1.07	(0.90-1.28)		418	1.09	(0.88-1.36)	
	30-34 y	455	1.13	(0.95-1.35)		416	1.19	(0.94-1.52)	
	35-39 y	179	1.06	(0.85-1.33)		158	1.13	(0.83-1.52)	
	40+ y	27	0.93	(0.60-1.44)		20	0.85	(0.49-1.48)	
Age of father	<25 y	80	1.00		0.97	80	1.00		0.58
	25-29 y	297	0.91	(0.69-1.19)		295	0.84	(0.62-1.12)	
	30-34 y	437	0.91	(0.70-1.19)		429	0.78	(0.57-1.07)	
	35-39 y	270	0.91	(0.69-1.21)		262	0.76	(0.54-1.06)	
	40+ y	134	0.93	(0.68-1.28)		131	0.81	(0.56-1.18)	
Birth weight	<2500 g	65	0.90	(0.68-1.18)	0.006	55	0.72	(0.52-1.00)	<0.001
	2500-3999 g	1,105	1.00			1,004	1.00		
	≧4000 g	147	1.36	(1.12-1.66)		138	1.42	(1.16-1.75)	
Multiple birth	singleton	1,312	1.00		0.004	1,154	1.00		0.003
	multiple	48	1.68	(1.20-2.36)		43	1.94	(1.27-2.96)	
Nationality of mother	Swiss	1,007	1.00		0.24	885	1.00		0.36
	other	350	0.92	(0.80-1.06)		312	0.93	(0.79-1.09)	

[a]Odds ratio of raw conditional logistic regression models; [b]95% confidence interval; [c]P-value of likelihood ratio test comparing model with and without a given perinatal characteristic; [d]Odds ratio of conditional logistic regression models adjusting for birth order, interval to next older sibling, age of mother, age of father, birth weight, multiple birth, and nationality of mother.

Table S8. Associations between perinatal characteristics and childhood leukemia, diagnosed at age 0-15 years, excluding multiple births

Exposure	Categories	Crude Cases	Crude OR[a]	Crude 95% CI[b]	Crude p-value[c]	Adjusted Cases	Adjusted OR[d]	Adjusted 95% CI[b]	Adjusted p-value[c]
Birth order	1st born	565	1.00		0.98	543	1.00		0.67
	2nd	457	0.99	(0.86-1.13)		441	0.58	(0.23-1.49)	
	3rd	160	1.01	(0.83-1.23)		154	0.58	(0.22-1.51)	
	4th or later	53	1.05	(0.76-1.43)		51	0.61	(0.23-1.64)	
Interval to next older sibling	1st born	549	1.00		0.60	548	1.00		0.39
	1-2 y	384	1.00	(0.86-1.15)		382	1.65	(0.65-4.24)	
	3-4 y	156	0.99	(0.81-1.20)		156	1.66	(0.64-4.30)	
	5+ y	104	1.17	(0.92-1.48)		103	1.95	(0.74-5.13)	
Age of mother	<25 y	224	1.00		0.70	185	1.00		0.85
	25-29 y	474	1.07	(0.90-1.27)		416	1.08	(0.87-1.33)	
	30-34 y	446	1.11	(0.92-1.32)		407	1.14	(0.90-1.46)	
	35-39 y	175	1.05	(0.84-1.31)		155	1.12	(0.83-1.51)	
	40+ y	35	1.29	(0.86-1.92)		26	1.21	(0.73-2.03)	
Age of father	<25 y	76	1.00		0.99	76	1.00		0.86
	25-29 y	296	0.94	(0.71-1.25)		294	0.88	(0.65-1.19)	
	30-34 y	436	0.95	(0.72-1.25)		430	0.84	(0.61-1.16)	
	35-39 y	270	0.97	(0.73-1.29)		262	0.83	(0.59-1.16)	
	40+ y	130	0.97	(0.71-1.34)		127	0.83	(0.56-1.21)	
Birth weight	<2500 g	48	0.65	(0.48-0.89)	<0.001	41	0.65	(0.46-0.91)	<0.001
	2500-3999 g	1,115	1.00			1,009	1.00		
	≧4000 g	148	1.33	(1.10-1.62)		139	1.39	(1.13-1.71)	
Nationality of mother	Swiss	998	1.00		0.32	874	1.00		0.44
	other	353	0.93	(0.81-1.07)		315	0.94	(0.80-1.10)	

[a]Odds ratio of raw conditional logistic regression models; [b]95% confidence interval; [c]P-value of likelihood ratio test comparing model with and without a given perinatal characteristic; [d]Odds ratio of conditional logistic regression models adjusting for birth order, interval to next older sibling, age of mother, age of father, birth weight, multiple birth, and nationality of mother.

Table S9. Associations between perinatal characteristics and ALL, diagnosed at age 0-15 years, excluding multiple births

		Crude				Adjusted			
**Exposure**	**Categories**	**Cases**	**OR[a]**	**95% CI[b]**	**p-value[c]**	**Cases**	**OR[d]**	**95% CI[b]**	**p-value[c]**
Birth order	1st born	458	1.00		0.76	438	1.00		0.48
	2nd	382	1.06	(0.91-1.23)		368	0.61	(0.21-1.75)	
	3rd	126	0.97	(0.78-1.21)		121	0.54	(0.19-1.59)	
	4th or later	41	0.91	(0.64-1.30)		39	0.52	(0.17-1.57)	
Interval to next older sibling	1st born	443	1.00		0.76	442	1.00		0.54
	1-2 y	320	1.04	(0.89-1.22)		318	1.68	(0.59-4.83)	
	3-4 y	125	1.02	(0.82-1.27)		125	1.66	(0.57-4.83)	
	5+ y	82	1.15	(0.89-1.50)		81	1.94	(0.66-5.73)	
Age of mother	<25 y	178	1.00		0.37	144	1.00		0.41
	25-29 y	382	1.10	(0.90-1.33)		335	1.13	(0.89-1.44)	
	30-34 y	371	1.20	(0.98-1.47)		341	1.30	(0.99-1.70)	
	35-39 y	146	1.07	(0.84-1.36)		127	1.19	(0.85-1.66)	
	40+ y	28	1.31	(0.84-2.05)		19	1.15	(0.64-2.07)	
Age of father	<25 y	61	1.00		0.90	61	1.00		0.84
	25-29 y	233	0.94	(0.69-1.29)		231	0.87	(0.62-1.21)	
	30-34 y	363	1.02	(0.76-1.39)		361	0.87	(0.61-1.23)	
	35-39 y	221	1.00	(0.73-1.37)		214	0.81	(0.56-1.18)	
	40+ y	101	0.94	(0.66-1.35)		99	0.80	(0.52-1.22)	
Birth weight	<2500 g	41	0.70	(0.50-0.98)	<0.001	36	0.73	(0.51-1.05)	<0.001
	2500-3999 g	900	1.00			811	1.00		
	≧4000 g	126	1.45	(1.17-1.79)		119	1.52	(1.21-1.91)	
Nationality of mother	Swiss	819	1.00		0.36	716	1.00		0.60
	other	283	0.93	(0.79-1.09)		250	0.95	(0.80-1.14)	

[a]Odds ratio of raw conditional logistic regression models; [b]95% confidence interval; [c]P-value of likelihood ratio test comparing model with and without a given perinatal characteristic; [d]Odds ratio of conditional logistic regression models adjusting for birth order, interval to next older sibling, age of mother, age of father, birth weight, multiple birth, and nationality of mother.

Table S10. Associations between perinatal characteristics and childhood leukemia, diagnosed at age 0-4 years, excluding multiple births

		Crude				Adjusted			
Exposure	Categories	Cases	OR[a]	95% CI[b]	p-value[c]	Cases	OR[d]	95% CI[b]	p-value[c]
Birth order	1st born	320	1.00		0.19	318	1.00		0.36
	2nd	232	0.85	(0.71-1.03)		230	0.38	(0.09-1.64)	
	3rd	77	0.84	(0.63-1.10)		76	0.36	(0.08-1.58)	
	4th or later	23	0.71	(0.45-1.13)		23	0.31	(0.07-1.40)	
Interval to next older sibling	1st born	321	1.00		0.24	320	1.00		0.47
	1-2 y	195	0.82	(0.67-1.00)		194	2.08	(0.49-8.94)	
	3-4 y	82	0.89	(0.68-1.17)		82	2.30	(0.53-9.98)	
	5+ y	51	0.96	(0.69-1.34)		51	2.50	(0.57-11.10)	
Age of mother	<25 y	109	1.00		0.84	93	1.00		0.37
	25-29 y	244	1.00	(0.78-1.28)		220	1.11	(0.83-1.50)	
	30-34 y	245	1.09	(0.85-1.40)		224	1.34	(0.96-1.87)	
	35-39 y	108	1.06	(0.78-1.42)		95	1.40	(0.93-2.10)	
	40+ y	22	1.24	(0.74-2.08)		15	1.22	(0.62-2.42)	
Age of father	<25 y	38	1.00		0.97	38	1.00		0.76
	25-29 y	156	0.96	(0.65-1.43)		154	0.93	(0.61-1.42)	
	30-34 y	244	0.99	(0.67-1.45)		239	0.89	(0.57-1.38)	
	35-39 y	148	0.91	(0.61-1.36)		142	0.78	(0.48-1.25)	
	40+ y	77	0.99	(0.63-1.55)		74	0.85	(0.50-1.44)	
Birth weight	<2500 g	31	0.76	(0.52-1.13)	0.041	26	0.76	(0.49-1.17)	0.010
	2500-3999 g	615	1.00			547	1.00		
	≧4000 g	78	1.33	(1.01-1.73)		74	1.49	(1.12-1.98)	
Nationality of mother	Swiss	522	1.00		0.82	462	1.00		0.72
	other	205	0.98	(0.81-1.18)		185	1.04	(0.84-1.28)	

[a]Odds ratio of raw conditional logistic regression models; [b]95% confidence interval; [c]P-value of likelihood ratio test comparing model with and without a given perinatal characteristic; [d]Odds ratio of conditional logistic regression models adjusting for birth order, interval to next older sibling, age of mother, age of father, birth weight, multiple birth, and nationality of mother.

Table S11. Associations between perinatal characteristics and AML, diagnosed at age 0-15 years, excluding multiple births

Exposure	Categories	Crude Cases	Crude OR[a]	Crude 95% CI[b]	Crude p-value[c]	Adjusted Cases	Adjusted OR[d]	Adjusted 95% CI[b]	Adjusted p-value[c]
Birth order	1st born	72	1.00		0.016	71	1.00		0.071
	2nd	52	0.71	(0.48-1.04)		51	0.78	(0.07-8.18)	
	3rd	26	1.23	(0.73-2.07)		25	1.32	(0.12-14.76)	
	4th or later	9	2.55	(1.09-5.98)		9	2.46	(0.22-27.60)	
Interval to next older sibling	1st born	72	1.00		0.44	72	1.00		0.73
	1-2 y	43	0.78	(0.52-1.19)		43	0.91	(0.09-9.51)	
	3-4 y	24	0.94	(0.56-1.56)		24	0.99	(0.09-10.58)	
	5+ y	17	1.32	(0.70-2.47)		17	1.38	(0.12-15.35)	
Age of mother	<25 y	25	1.00		0.25	22	1.00		0.14
	25-29 y	73	1.44	(0.87-2.37)		65	1.74	(0.91-3.33)	
	30-34 y	52	0.94	(0.56-1.60)		46	1.14	(0.55-2.37)	
	35-39 y	19	1.22	(0.62-2.40)		18	1.32	(0.53-3.30)	
	40+ y	5	1.60	(0.53-4.87)		5	3.17	(0.75-13.36)	
Age of father	<25 y	10	1.00		0.20	10	1.00		0.22
	25-29 y	46	1.04	(0.49-2.24)		46	0.74	(0.31-1.79)	
	30-34 y	53	0.70	(0.33-1.48)		51	0.47	(0.18-1.18)	
	35-39 y	29	0.71	(0.32-1.62)		28	0.46	(0.17-1.29)	
	40+ y	21	1.22	(0.51-2.94)		21	0.67	(0.22-2.03)	
Birth weight	<2500 g	4	0.36	(0.13-1.01)	0.051	3	0.28	(0.08-0.95)	0.045
	2500-3999 g	154	1.00			141	1.00		
	≧4000 g	13	0.70	(0.37-1.29)		12	0.74	(0.38-1.47)	
Nationality of mother	Swiss	123	1.00		0.58	108	1.00		0.88
	other	51	1.11	(0.76-1.63)		48	1.03	(0.67-1.60)	

[a]Odds ratio of raw conditional logistic regression models; [b]95% confidence interval; [c]P-value of likelihood ratio test comparing model with and without a given perinatal characteristic; [d]Odds ratio of conditional logistic regression models adjusting for birth order, interval to next older sibling, age of mother, age of father, birth weight, multiple birth, and nationality of mother.

Table S12. Associations between perinatal characteristics and childhood leukemia, diagnosed at age 0-15 years, cases born 1980-1989

Exposure	Categories	Cases	Adjusted OR[a]	95% CI[b]	p-value[c]
Birth order	1st born	116	1.00		0.52
	2nd	115	1.86	(0.52-6.65)	
	3rd	43	1.66	(0.44-6.23)	
	4th or later	18	2.51	(0.60-10.44)	
Interval	1st born	120	1.00		0.87
	1-2 y	105	0.57	(0.16-2.05)	
	3-4 y	39	0.57	(0.15-2.10)	
	5+ y	28	0.55	(0.14-2.13)	
Age mother	<25 y	53	1.00		0.96
	25-29 y	125	1.16	(0.78-1.71)	
	30-34 y	85	1.12	(0.70-1.78)	
	35-39 y	26	1.04	(0.54-2.02)	
	40+ y	3	0.99	(0.24-4.04)	
Age father	<25 y	17	1.00		0.88
	25-29 y	88	1.25	(0.69-2.27)	
	30-34 y	108	1.28	(0.68-2.41)	
	35-39 y	54	1.40	(0.71-2.77)	
	40+ y	25	1.49	(0.67-3.34)	
Birth weight	<2500 g	9	0.60	(0.28-1.29)	0.066
	2500-3999 g	249	1.00		
	≧4000 g	34	1.48	(0.97-2.27)	
Multiple birth	singleton	285	1.00		0.37
	multiple	7	1.60	(0.59-4.31)	
Nationality of mother	Swiss	257	1.00		0.57
	other	35	0.89	(0.58-1.35)	

[a]Adjusted OR odds ratio of conditional logistic regression models adjusting for birth order, interval to next older sibling, age of mother, age of father, birth weight, multiple birth, and nationality of mother; [b]95% confidence interval; [c]P-value likelihood ratio test comparing model with and without a given perinatal characteristic.

Table S13. Associations between perinatal characteristics and childhood leukemia, diagnosed at age 0-15 years, cases born 1990-1999

**Exposure**	**Categories**	**Cases**	**Adjusted OR[a]**	**95% CI[b]**	**p-value[c]**
Birth order	1st born	204	1.00		0.32
	2nd	173	1.15	(0.42-3.13)	
	3rd	67	1.34	(0.47-3.81)	
	4th or later	13	0.74	(0.23-2.34)	
Interval	1st born	212	1.00		0.54
	1-2 y	153	0.86	(0.32-2.35)	
	3-4 y	56	0.80	(0.28-2.23)	
	5+ y	36	1.13	(0.39-3.29)	
Age mother	<25 y	80	1.00		0.66
	25-29 y	159	0.94	(0.67-1.32)	
	30-34 y	162	1.17	(0.80-1.70)	
	35-39 y	49	1.14	(0.70-1.86)	
	40+ y	7	1.14	(0.46-2.86)	
Age father	<25 y	37	1.00		0.23
	25-29 y	126	0.78	(0.50-1.22)	
	30-34 y	164	0.62	(0.39-1.01)	
	35-39 y	89	0.57	(0.34-0.96)	
	40+ y	41	0.64	(0.35-1.16)	
Birth weight	<2500 g	24	0.73	(0.43-1.24)	0.013
	2500-3999 g	379	1.00		
	≧4000 g	54	1.59	(1.14-2.22)	
Multiple birth	singleton	438	1.00		0.004
	multiple	19	2.98	(1.44-6.17)	
Nationality of mother	Swiss	346	1.00		0.070
	other	111	0.78	(0.60-1.02)	

[a]Adjusted OR odds ratio of conditional logistic regression models adjusting for birth order, interval to next older sibling, age of mother, age of father, birth weight, multiple birth, and nationality of mother; [b]95% confidence interval; [c]P-value likelihood ratio test comparing model with and without a given perinatal characteristic.

Table S14. Associations between perinatal characteristics and childhood leukemia, diagnosed at age 0-15 years, cases born 2000-2009

Exposure	Categories	Cases	Adjusted OR[a]	95% CI[b]	p-value[c]
Birth order	1st born	176	1.00		0.14
	2nd	132	0.86	(0.21-3.44)	
	3rd	35	0.63	(0.15-2.65)	
	4th or later	17	1.41	(0.30-6.53)	
Interval	1st born	179	1.00		0.93
	1-2 y	106	1.18	(0.29-4.74)	
	3-4 y	46	1.16	(0.28-4.76)	
	5+ y	29	1.36	(0.32-5.76)	
Age mother	<25 y	44	1.00		0.65
	25-29 y	106	1.10	(0.70-1.73)	
	30-34 y	136	1.13	(0.69-1.84)	
	35-39 y	67	1.15	(0.65-2.01)	
	40+ y	7	0.62	(0.24-1.62)	
Age father	<25 y	22	1.00		0.38
	25-29 y	63	0.60	(0.33-1.11)	
	30-34 y	133	0.72	(0.38-1.34)	
	35-39 y	94	0.59	(0.30-1.13)	
	40+ y	48	0.65	(0.32-1.32)	
Birth weight	<2500 g	17	0.64	(0.36-1.13)	0.14
	2500-3999 g	304	1.00		
	≧4000 g	39	1.26	(0.85-1.86)	
Multiple birth	singleton	345	1.00		0.15
	multiple	15	1.71	(0.84-3.49)	
Nationality of mother	Swiss	218	1.00		0.25
	other	142	1.17	(0.90-1.52)	

[a]Adjusted OR odds ratio of conditional logistic regression models adjusting for birth order, interval to next older sibling, age of mother, age of father, birth weight, multiple birth, and nationality of mother; [b]95% confidence interval; [c]P-value likelihood ratio test comparing model with and without a given perinatal characteristic.

Figures

Figure S1. Correlation matrix of the birth characteristics. Spearman's rank correlation coefficients (ρ) of the variables included in the fully adjusted conditional logistic regression models treated in highest resolution (continuous or ordinal) using complete observations only.

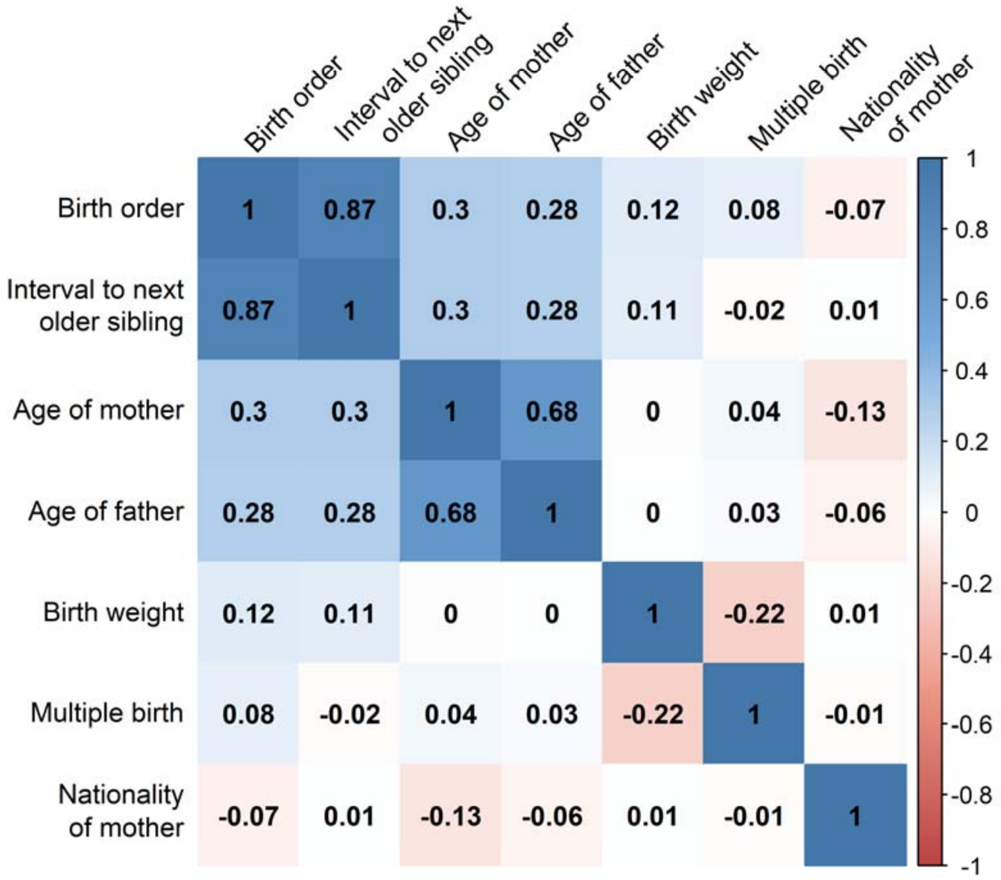

Supplement: Supplementary file 1 — Supplementary file1 (PDF 282 kb) [file 10552_2021_1423_MOESM1_ESM.pdf]
